# AOHE: manuscript AOHE-D-16-00564 paroxysmal nocturnal hemoglobinuria with autoimmune hemolytic anemia following eculizumab therapy—with large granular lymphocytic leukemia

**DOI:** 10.1007/s00277-016-2752-5

**Published:** 2016-07-23

**Authors:** Nathan Visweshwar, Michael Jaglal, Cassie Booth, Patrick Griffin, Damian Laber

**Affiliations:** 1 Department of Hematology, University of South Florida, 13330 USF Laurel Drive Tampa, Tampa, FL 33612 USA; 2 Moffitt Cancer Center, Tampa, FL USA; 3 Tampa General Hospital, Tampa, FL USA

Dear Editor,

A 49-year-old Turkish male presented with intermittent passage of dark-colored urine since 2007. Flow cytometry demonstrated absence of CD55 and CD59 antigens, consistent with a diagnosis of paroxysmal nocturnal hemoglobinuria (PNH). He initially responded to eculizumab, with improvement in anemia and resolution of hemoglobinuria. In 2009, the patient developed Coombs positive autoimmune hemolytic anemia (AIHA), which responded to steroids. Since he continued to require high doses of prednisone, splenectomy was performed in November 2009. Unfortunately, his AIHA persisted and he was treated with four weekly doses of rituximab 375 mg/m^2^ in June 2010. At that time, fluorescently labeled aerolysin (FLAER) labeling on leukocytes failed to demonstrate PNH, so treatment with eculizumab was discontinued. In April 2014, the patient presented with septic shock in the setting of asplenia. He developed acral ischemic necrosis, requiring amputation of multiple fingers as well as his left leg below the knee. With appropriate resuscitation and antibiotic therapy, he was able to recover and he eventually regained independence with the aid of prosthesis. In February 2016, his complete blood count showed white blood cells 15,170/mcL, with 66 % lymphocytes, hemoglobin 9.2 g/dl, and platelets 510,000/mcL. His peripheral blood smear revealed a predominance of large granular lymphocytes (LGL), which were CD8 and CD57 positive by flow cytometry (Figs. 1 and 2). Additional studies revealed clonal rearrangement of both beta and gamma T cell receptor genes, as well as a CD4:CD8 ratio of 0.1. He continues to require prednisone for AIHA.

**Fig. 1:**
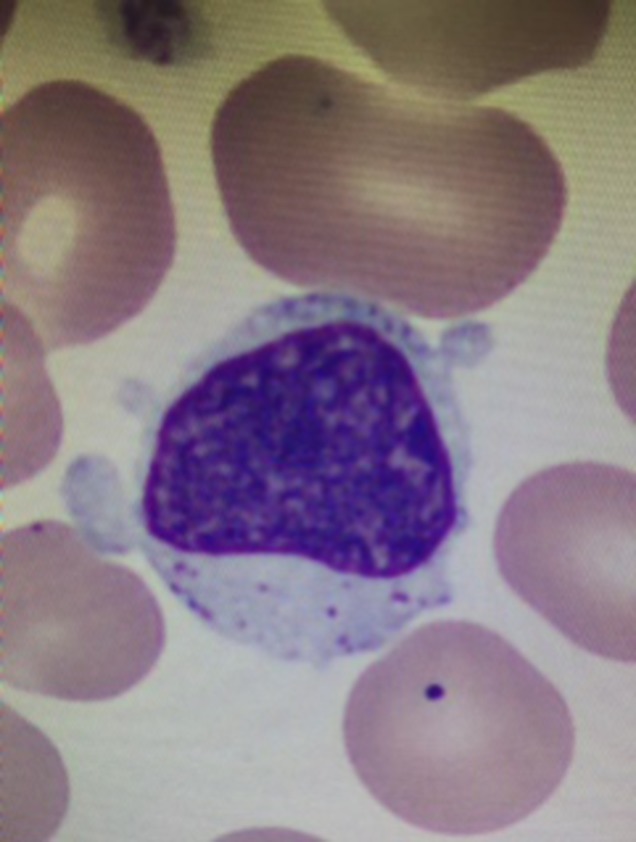
Peripheral blood smear showing a large granular lymphocyte and a Howell-Jolly body (status post splenectomy)

**Fig. 2:**
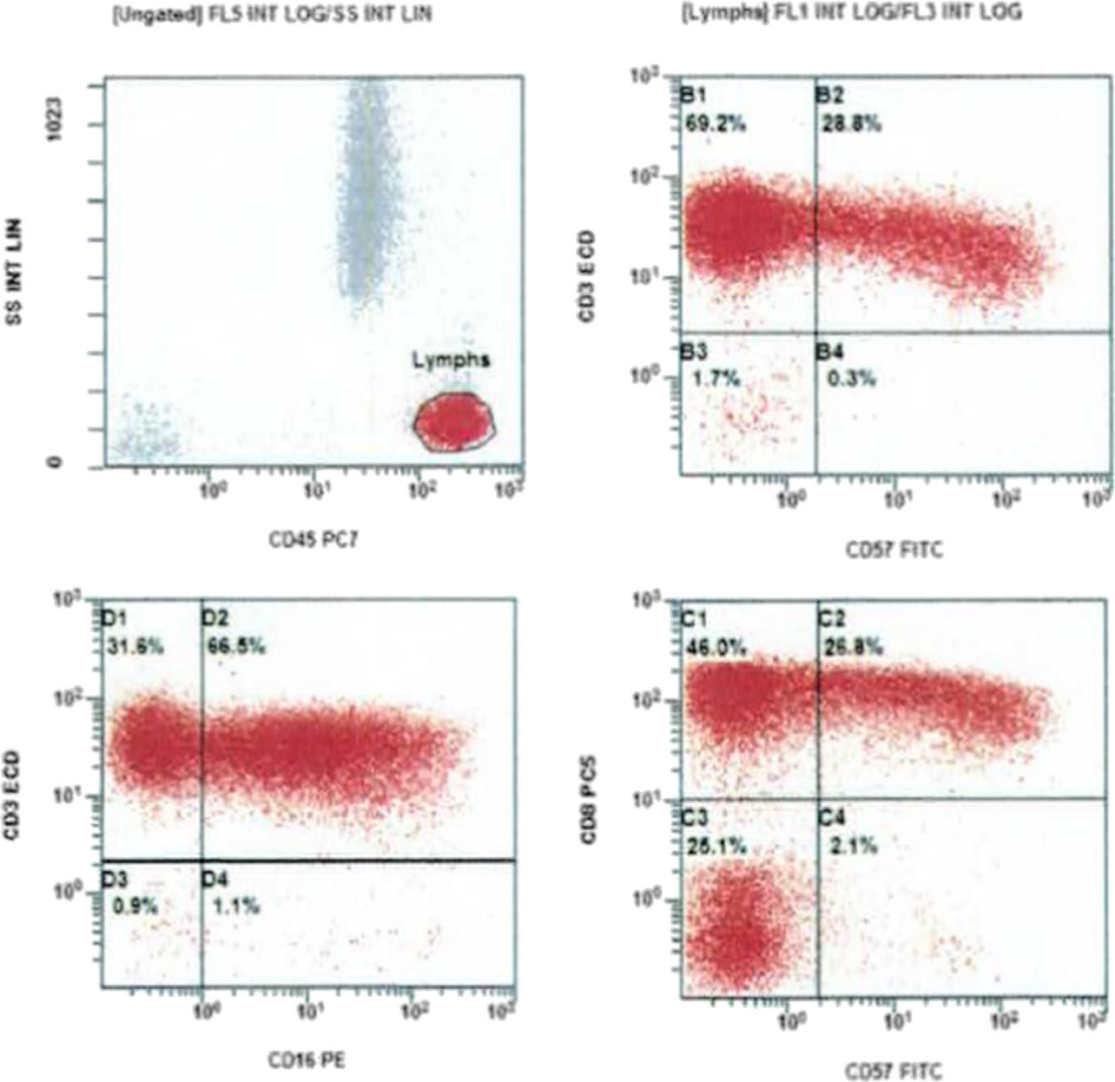
Flow cytometry demonstrating a lymphocyte population with expression of both CD8 and CD57

Paroxysmal nocturnal hemoglobinuria (PNH) is an acquired clonal disorder caused by somatic mutation of the X-linked gene, PIG-A, which results in the deficiency of glycosylphosphatidylinositol (GPI) [1]. Deficiency of specific GPI anchored proteins, CD55 and CD59, renders red cells exquisitely sensitive to complement mediated destruction, leading to unabated intravascular hemolysis [2]. Complement blockade through the anti-C5 antibody, eculizumab, initially prevents hemolysis. However, over a period of time, a large number of C3 antigens accumulate [3]. C3 rich red cells with abnormal GPI molecules sensitize the immune system, resulting in T cell recognition and opsonization by macrophages of the reticuloendothelial system [4]. This phenomenon results in extravascular hemolysis with Coombs positivity in PNH patients on eculizumab [5]. Recent reports indicate that GPI-specific CD8+ T cells, which have been identified in PNH patients, spare selectively GPI-negative stem cells, thus enabling them to re-populate the marrow of a patient who would otherwise have aplastic anemia [6]. T cell receptor beta (TCR-beta) clones of the CD8+ CD57+ T cell population are frequently deranged in PNH, but not in healthy controls [7]. T cell clones bearing a set of highly homologous TCR-beta molecules in PNH suggest an immune process driven by non-peptide antigen, as patients do not share identical HLA alleles. In PNH patients, the presence of CD8 (+) T cells reactive against antigen-presenting cells (APCs) loaded with GPI are seen in higher numbers than in healthy controls [8]. To our knowledge, this is the first documented case of PNH with clonally mutated large granular lymphocytic leukemia with beta and gamma gene rearrangement, reported in the literature.

## References

[1] Yamada N (1995). Somatic mutations of the PIG-A gene found in Japanese patients with paroxysmal nocturnal hemoglobinuria. Blood.

[2] Navenot JM (1993). Rapid diagnosis of paroxysmal nocturnal hemoglobinuria by gel test agglutination. Rev Fr Transfus Hemobiol.

[3] Nakayama H (2016). Eculizumab dosing intervals longer than 17 days may be associated with greater risk of breakthrough hemolysis in patients with paroxysmal nocturnal hemoglobinuria. Biol Pharm Bull.

[4] Risitano AM (2009). Complement fraction 3 binding on erythrocytes as additional mechanism of disease in paroxysmal nocturnal hemoglobinuria patients treated by eculizumab. Blood.

[5] Hochsmann B (2012). Paroxysmal nocturnal haemoglobinuria treatment with eculizumab is associated with a positive direct antiglobulin test. Vox Sang.

[6] Luzzatto L (2016) Recent advances in the pathogenesis and treatment of paroxysmal nocturnal hemoglobinuria. F1000Res p 5. doi:10.12688/f1000research.7288.1 10.12688/f1000research.7288.1 PMC4765720 26962442

[7] Gargiulo L (2007). Highly homologous T-cell receptor beta sequences support a common target for autoreactive T cells in most patients with paroxysmal nocturnal hemoglobinuria. Blood.

[8] Gargiulo L (2013). Glycosylphosphatidylinositol-specific, CD1d-restricted T cells in paroxysmal nocturnal hemoglobinuria. Blood.

